# Mitochondrial Genome Analysis of Philippine Eagles (*Pithecophaga jefferyi* Ogilvie‐Grant 1896) From the Greater Mindanao Faunal Region

**DOI:** 10.1002/ece3.72572

**Published:** 2025-12-04

**Authors:** Michael G. Bacus, Paul Lorenzo A. Gaite, Joan T. Acaso, Joshua M. Cambronero, Gerry N. Ramos, April Mae M. Numeron, Dominic Tadena, Christian C. Labrador, Mae A. Responte, Lief Erikson D. Gamalo, Jayson C. Ibañez, Lyre Anni E. Murao

**Affiliations:** ^1^ Philippine Genome Center Mindanao University of the Philippines Mindanao Davao City Philippines; ^2^ Department of Biological Sciences and Environmental Studies, College of Science and Mathematics University of the Philippines Mindanao Davao City Philippines; ^3^ Philippine Eagle Foundation Philippine Eagle Center Davao City Philippines; ^4^ Wildlife‐Human Interaction Studies, Ecological Research and Biodiversity Conservation Laboratory University of the Philippines Mindanao Davao City Philippines

**Keywords:** conservation breeding, genetic diversity, mitogenome, phylogenetics

## Abstract

The population of the endemic and critically endangered Philippine eagle ( *Pithecophaga jefferyi* Ogilvie‐Grant 1896) has dramatically declined, which may lead to reduced genetic diversity and increased inbreeding and extinction risk. To address the concern, there is an urgent need for genetic data to guide conservation breeding, preserve the genetic diversity of the species, and improve reintroduction outcomes. In this study, we performed mitochondrial genome sequencing and assessed the genetic diversity of Philippine eagles. Analysis of partial mitogenomes (12 protein‐coding genes, 17 tRNAs, and 16S rRNA) revealed a very low overall nucleotide diversity (*π* = 0.00054 ± 0.00030) among 32 eagles from the Greater Mindanao Faunal Region. A higher number of haplotypes were observed in the eagles bred in captivity at the Philippine Eagle Center (*h* = 17) compared to the wild captives (*h* = 13), but both population subsets had comparable nucleotide and haplotype diversity. At least two distinct Philippine eagle subpopulations based on maternal ancestry were identified, although there is generally low genetic differentiation based on *F*_*ST*_ analysis. Furthermore, isolation by distance analysis (geographic isolation) showed a weak positive correlation between the genetic distance and the geographic distance of the eagle population. This suggests possible historical connectivity or limited influence of geographic separation on the genetic differentiation in Philippine eagles. Nevertheless, three genetically distant haplotypes and several eagles from KBA dyads with moderate levels of genetic differentiation were identified, representing the existence of genetically useful breeding stocks that can be prioritized in the current conservation program. Finally, phylogenetic analysis of Philippine eagles and other members of the family Accipitridae corroborates previous findings on the inclusion of the species in the subfamily Circaetinae and its monophyletic status. Altogether, we demonstrate the utility of mitogenomes in assessing genetic diversity and evolutionary relationships for species conservation.

## Graphical abstract

Mitogenome sequencing is a promising alternative for the genetic studies of animals. In this work, we examined the genetic diversity of the critically endangered and endemic Philippine eagles and their evolutionary relationship to other birds of prey. Our work provides important genetic information that can be used to enhance the current Philippine eagle captive breeding program to preserve the genetic diversity of the species and increase the success rate of species reintroduction in the wild.

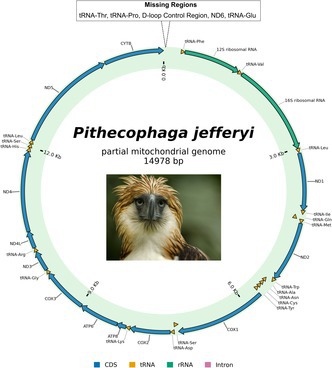

## 1 Introduction

The Philippine eagle ( *Pithecophaga jefferyi* Ogilvie‐Grant 1896) belongs to the Family Accipitridae, a group composed of some of the most threatened bird species (McClure et al. 2023). The species is endemic to the Philippines with population records from the three large islands of the Greater Mindanao Faunal Region (Mindanao, Leyte and Samar), and in the eastern portion of Luzon island (Kennedy 2000). Being a forest‐dependent raptor, the eagle population's major threats are deforestation throughout its range, hunting and climate change (Ibañez et al. 2016; BirdLife International 2018). The International Union for the Conservation of Nature (IUCN) and the Philippine Department of Environment and Natural Resources (DENR) consider the *P. jefferyi* as a Critically Endangered species (BirdLife International 2018; DENR Administrative Order 2019–09, n.d.), with an estimated population of 392 potentially breeding pairs (Sutton et al. 2023). The continued decline of the eagle's population in the wild is further complicated by its late maturation, slow breeding cycle, and high mate and nest fidelity (Ibañez et al. 2016; Miranda Jr et al. 2000), which altogether limits the ability of the wild population to recover.

The population decline of the species could result in a genetic bottleneck, a stochastic event that decreases genetic variation (Nei et al. 1975). Genetic bottlenecks increase inbreeding rates which decrease overall fitness and survival of populations in the wild (Frankham 1998; Keller and Waller 2002). A previous study using the mitochondrial control region of 19 Philippine eagles rescued from the wild, together with 3 captive‐bred individuals, showed high genetic diversity comparable to non‐threatened raptor species (Luczon et al. 2014). However, the same study detected indicators of possible recent genetic bottlenecks (Luczon et al. 2014). Despite the increasing concern for highly threatened species, information on the genetic background of the species is still incomplete. These concerns underscore the need for more genetic studies that could provide information instrumental for decision‐making for the eagle's conservation and management.

There is an urgent need for effective conservation breeding efforts that lead to releases of suitably reared Philippine eagles to repopulate vacant habitats and save the species from extinction (Collar and Butchart 2014). To address this concern, the Philippine Eagle Foundation (PEF), a private and non‐profit organization, works at the forefront of conservation breeding and in the protection of rainforest habitats for the species through holistic conservation efforts. Its office base is located at the Philippine Eagle Center (PEC) in Davao City, Philippines, and functions both as a conservation breeding and species rehabilitation facility for eagles rescued from the wild. PEF also employs both natural pairing techniques and cooperative artificial insemination in breeding eagles in captivity. It has produced 30 captive‐bred Philippine eagles for the past 37 years since 1988, three of which have been released into the wild (Bastian Jr et al. 2007; Harder et al. 2006). In 2023, a new conservation breeding facility was established to isolate and optimize the productivity of captive birds in Mt. Apo, a pristine forest habitat away from human disturbances and emerging zoonotic and bird infectious diseases.

Conservation efforts could benefit from genetic information to improve captive breeding, and consequently, species reintroduction outcomes (Sato et al. 2017). However, there is currently a lack of genetic information for the species that can be used to inform pairing attempts (for natural pairs) and artificial insemination (for imprinted birds). Furthermore, it is still unclear whether captive breeding efforts undertaken by PEF, including the currently available eagle breeding stocks, are sufficient to maintain genetic diversity in captive‐bred eagles. This concern underscores the need for genetic data to support captive breeding efforts and facilitate population studies to better understand the evolutionary history of the species.

The use of mitochondrial DNA sequences presents a promising alternative to address the lack of genetic data for Philippine eagles. Due to its shorter genome size, maternal inheritance, and relatively high mutation rate, mitochondrial DNA is commonly used in species barcoding and population genetic studies in animals, including avian species (Xing et al. 2025). A previous study utilized a single locus, the mitochondrial control region, to initially assess the genetic diversity of the species (Luczon et al. 2014). With the availability of next‐generation sequencing technologies, it is now possible to sequence and assemble animal mitochondrial genomes (mitogenomes). Thus, this study employed mitochondrial DNA sequencing and comparatively assessed the genetic diversity among wild captives (eagles rescued from the wild) and those bred in captivity (both from natural pairs and artificial insemination). We further performed phylogenetic analysis to understand the evolutionary relationship of the species with other accipitrids. Altogether, the new genetic information provided by this study could prove useful in improving the current captive breeding programs and conservation efforts for Philippine eagles.

## 2 Materials and Methods

### 2.1 Sample Origin

A total of 32 Philippine eagles currently housed at PEF with residual blood samples were included in this study. The residual blood samples in EDTA‐containing collection tubes with volumes less than 1 mL were provided by the Philippine Eagle Center and transported to the Philippine Genome Center Mindanao laboratory for long‐term storage at −20°C. PEF through the Philippine Eagle Center is currently designated as the official conservation breeding facility for Philippine eagles. The PEF has a 10‐year Memorandum of Agreement (MOA) with the Philippine Department of Environment and Natural Resources (DENR) that provides the legal and ethical basis to undertake annual physical and medical screening for Philippine eagles as well as undertake genetic studies, species rehabilitation, captive breeding, and species reintroduction (MOA No. SENR067470).

**FIGURE 1:**
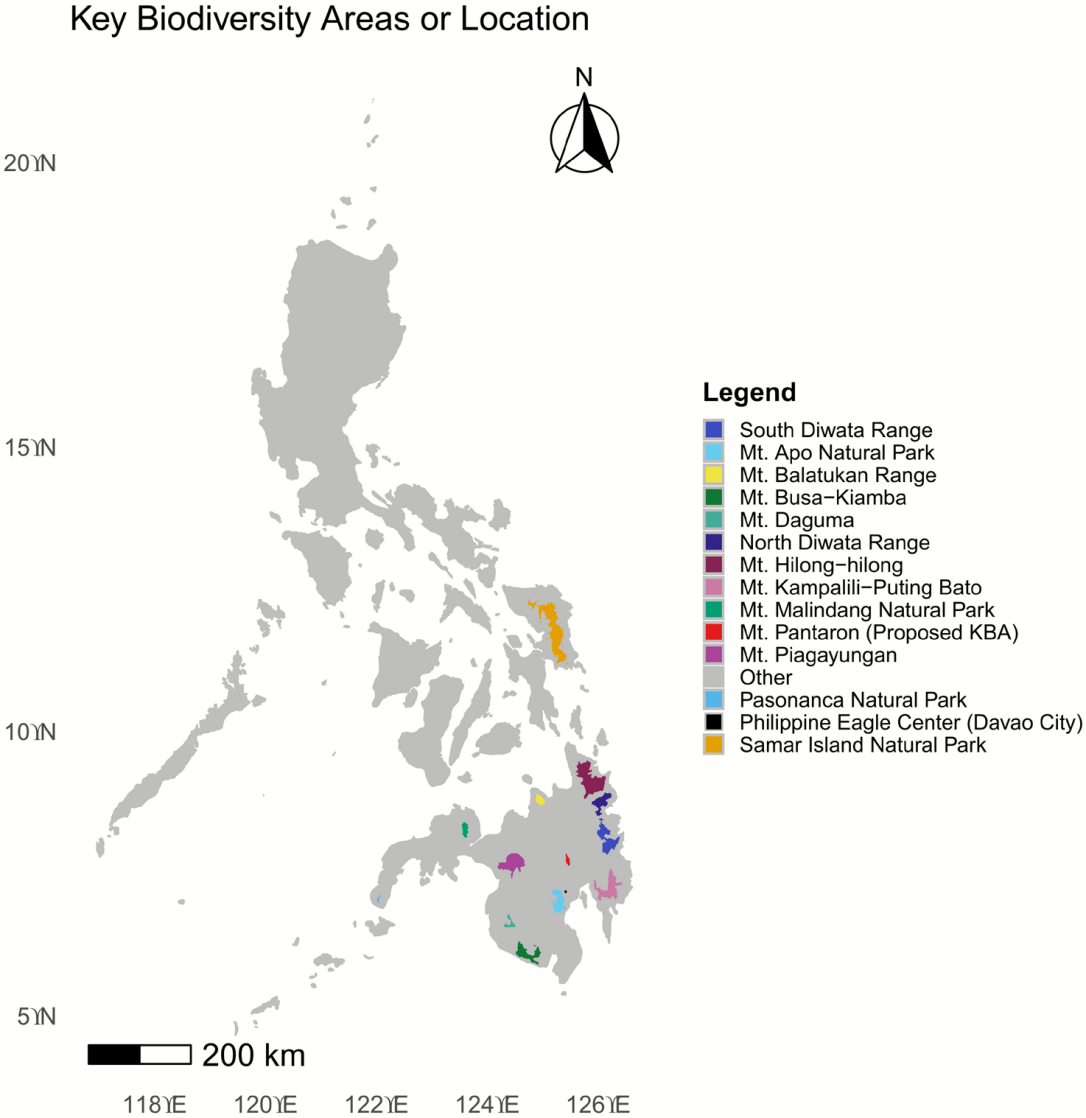
Geographic origin of the Philippine eagle individuals rescued from the wild and included in this study is highlighted in the map at the level of the key biodiversity area (KBA) or its known habitat. Mt. Pantaron is highlighted as it is a newly proposed KBA. All PEC captive‐bred eagles were born at the Philippine Eagle Center in Davao City, Philippines.

Of the 32 samples, 19 were from eagles rescued from the wild (9 males, 10 females) from 1984 to 2021, and herein referred to as wild captives. The remaining 13 eagles were all bred in captivity (9 males, 4 females), and herein referred to as PEC captive‐breds, with the oldest hatched in 1992 and the youngest hatched in 2015. The relevant metadata for each eagle which includes their names, sex, hatch date (for PEC captive‐breds), PEC admission age and year (for wild captives), and geographic origin at the region, province/city, and key biodiversity area level (KBA) (for wild captives) within the Greater Mindanao Faunal Region are all listed in Table A1. Figure 1 illustrates the geographic origin of the 32 individuals included in this study.

### 2.2 DNA Extraction and Mitogenome Enrichment

Total DNA was extracted from 10 μL blood samples using the Qiagen DNeasy blood and tissue kit (Qiagen, Germany) according to the manufacturer's instructions. DNA extracts were assessed for their concentration (ng/μL) and purity (A260/A280nm) using a UV–Vis spectrophotometer (Multiskan SkyHigh, Thermo Fisher Scientific). All 32 DNA extracts were stored at −20°C prior to use.

**FIGURE A1:**
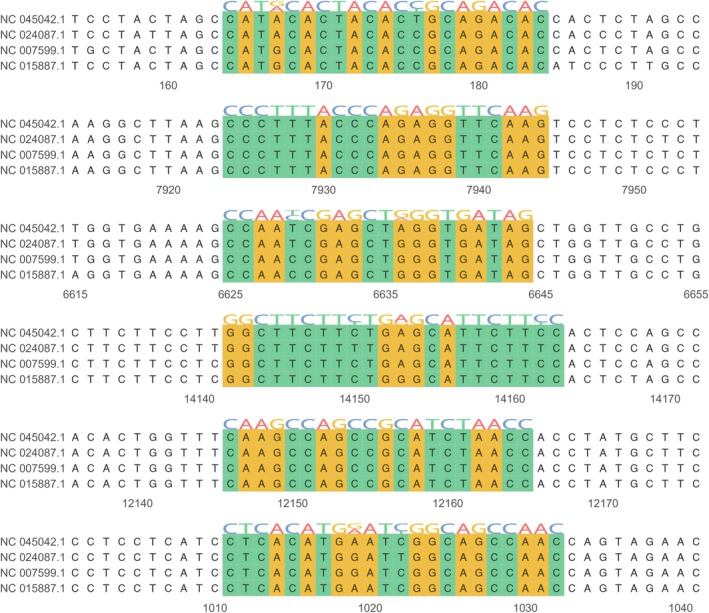
Primers designed to amplify more than 7 kb regions of the Philippine eagle mitogenome based on the conserved regions of reference mitogenome sequences of closely related eagle species available from GenBank. The multiple sequence alignment (MSA) was generated in MEGA X using the MUSCLE algorithm and sequences were manually checked to select for primers. The MSA shown here was generated using the ggmsa package in R for the following primers (from top to bottom): PEmtDNA_1FD, PEmtDNA_1RS, PEmtDNA_2FD, PEmtDNA_2RS, PEmtDNA_3FD, and PEmtDNA_3RS. The reverse primers (RS) are aligned using the reverse complement sequence.

We designed three sets of primers to enrich the Philippine eagle mitogenome via long range PCR each with an estimated amplicon size of more than 7 kb. Due to the absence of a reference mitogenome for the species, we used mitogenome sequences that were available from GenBank from closely related eagle species belonging to the same family Accipitridae namely, *Spilornis cheela* or crested serpent eagle (NC_015887), *Aquila chrysaetos* or golden eagle (NC_024087), *Aquila nipalensis* or steppe eagle (NC_045042), and *Nisaetus alboniger* or blyth's hawk‐eagle (NC_007599). Primers were designed to target conserved regions from the four reference mitogenome sequences (Figure A1). The resulting primer sequences, their position based on the *Spilornis cheela* mitogenome, and their *in silico* properties obtained from the Beacon Designer software (Free Edition, Premier Biosoft) are listed in Table A2.

Long range PCR was performed using the Phusion High Fidelity 2X master mix (New England Biolabs) with the following PCR mix composition: 5 μL of Phusion HF buffer, 0.5 μL of 10 mM dNTPs, 1.25 μL each of the 10 mM forward and reverse primers, 0.25 μL of Phusion Hot Start II DNA polymerase, 0.75 μL of DMSO, 10.8 μL of nuclease‐free water and 2 μL of at least 30 ng genomic DNA for a total volume of 20 μL per reaction. The thermal cycling conditions used were as follows: initial denaturation at 98°C for 2 min, 35 cycles of denaturation at 98°C for 10 s, annealing at 58°C (PEmtDNA 1FD/1RS, PEmtDNA 2FD/2RS) or 59°C (PEmtDNA 3FD/3RS) for 15 s, and extension at 72°C for 8 min. A final extension was added at 72°C for 10 min and the reaction was held at 4°C. All PCR products were visualized with agarose gel electrophoresis using a 1 μL volume in 2% agarose gel. The remaining PCR products were stored at −20°C prior to library preparation.

### 2.3 Library Preparation and DNA Sequencing

All 32 samples were processed for library preparation and Illumina sequencing. The three PCR amplicons for each sample were mixed using 10 μL volume each, for a total of 30 μL PCR amplicon pool. For each sample, 20 μL of the PCR amplicon pool was enzymatically tagmented with the use of the Enrichment Bead‐Linked Transposomes (EBLTS) (Illumina, USA), followed by ligation of dual index barcodes (Integrated DNA Technologies, Singapore) via PCR amplification with the Enhanced PCR Mix High Throughput (EPM HT) (Illumina, USA). Indexed libraries were pooled and assessed for fragment size distribution using Bioanalyzer DNA kit 1000 (Agilent, USA) and library concentration was estimated using Qubit HS DNA assay kit (Thermo Fisher Scientific). Libraries were then normalized and sequenced using NextSeq1000 SBS reagent cartridge (Illumina, USA) and P2 flow cell (Illumina, USA) in a paired‐end format with 200 cycles.

### 2.4 Mitogenome Assembly and Annotation

Raw paired‐end sequencing files in fastq format were subjected to quality control using FastQC v0.12.1 (Andrews et al. 2010). Paired reads were generated using Trimmomatic v0.39 (Bolger et al. 2014) and only Q30 reads were retained, followed by the removal of sequencing adapters and primer sequences using Cutadapt v3.5 (Martin 2011). Assembly of the mitochondrial genome was performed using NOVOPlasty v4.3.3 (Dierckxsens et al. 2017) and the Philippine eagle NADH dehydrogenase subunit 2 (ND2) gene as a seed (NCBI Accession No. AY987064.1). The resulting contigs were annotated initially using MitoAnnotator v4.09 (Iwasaki et al. 2013) and confirmed using the NCBI nucleotide and protein BLAST tools. All the assembled partial mitogenome sequences were deposited in GenBank and the respective accession numbers are listed in Table A1.

### 2.5 Multiple Sequence Alignment

Multiple sequence alignment (MSA) was performed using the MAFFT software (Katoh and Standley 2013) and a high‐accuracy global alignment method with 1000 iterations. The MSA was then processed using trimAl (Capella‐Gutiérrez et al. 2009) for complete deletion of sites with gaps. We generated two MSAs, the first one for all the 32 Philippine eagle partial mitogenome sequences generated in this study herein referred to as the Philippine Eagle dataset. The second MSA was generated from the protein‐coding genes (PCGs) of mitogenomes from accipitrids (Order Accipitriformes) retrieved from GenBank together with the samples sequenced in this study, herein referred to as the Accipitriformes dataset. All mitogenome sequences retrieved from GenBank are listed in Table A3.

### 2.6 Diversity Analysis

The Philippine Eagle dataset was used to calculate the nucleotide (π) and haplotype (h) diversity using DnaSP v.5.0 (Librado and Rozas 2009) with default parameters. A haplotype network was generated using TCS v.1.21 (Clement et al. 2000) and further annotated using tcsBU v.1.0 (Múrias dos Santos et al. 2016). The *F*_ST_ values for subpopulations based on the eagle's original habitat at the level of the key biodiversity area (KBA) were calculated using the *hierfstat* package in R (Goudet 2005) with the Nei87 method (Nei 1987) to estimate the degree of genetic differentiation. For *F*_*ST*_ analysis, the eagles bred in captivity at PEF were grouped as one subpopulation. Isolation by distance (IBD) of the Philippine eagle subpopulation was assessed to estimate the correlation of the pairwise genetic distance to the pairwise geographic distance using the Mantel test in the *Adegene*t package in R (Jombart 2008). Data on the geographic coordinates for each KBA were obtained from The Humanitarian Data Exchange platform (data deposited by the Philippine Department of Environment and Natural Resources—Biodiversity Management Bureau). Each KBA was represented by the centroid latitude and longitude calculated using the sf_centroid function and converted into UTM using the sf_transform function in the *sf* package in R (Pebesma 2018). The kml file for the Mt. Pantaron proposed KBA was manually obtained from Google Earth web and the centroid coordinates and UTM conversion performed using the *sf* package. The correlation plots with local densities of genetic and geographic distances were visualized using non‐parametric two‐dimensional kernel density estimates (KDE) (Jombart 2008).

### 2.7 Phylogenetic Analysis

Two phylogenetic trees were generated in this study. The first phylogenetic tree was constructed using the Philippine Eagle dataset to assess the evolutionary relationship of *P. jefferyi* haplotypes. The second phylogenetic tree was constructed using the Accipitriformes dataset to place the eagle samples in a broader phylogenetic context within the order Accipitriformes. In the second phylogenetic tree, representative species from the families Cathartidae, Pandionidae, and Sagittariidae were used as an outgroup. For both datasets, the best DNA substitution and site heterogeneity model was calculated in MEGA X (Kumar et al. 2018). The Bayesian phylogenetic analysis was performed in BEAST v.1.10.4 (Suchard and Lemey 2018) using the best‐fit model, empirical base frequencies, an uncorrelated relaxed molecular clock with lognormal distribution, and 100 million MCMC iterations with sampling every 10,000 trees. The estimated sampling size values (ESS) of all continuous parameters were assessed with Tracer v.1.7.2 (Rambaut et al. 2018) and the maximum clade credibility (MCC) tree was generated using TreeAnnotator v.1.10.4 with a 10% burn‐in. The resulting phylogenetic trees were visualized and annotated using the *ggtree* R package (Yu et al. 2017).

## 3 Results

### 3.1 Analysis of Partial Mitochondrial Genomes of Philippine Eagles

**FIGURE 2:**
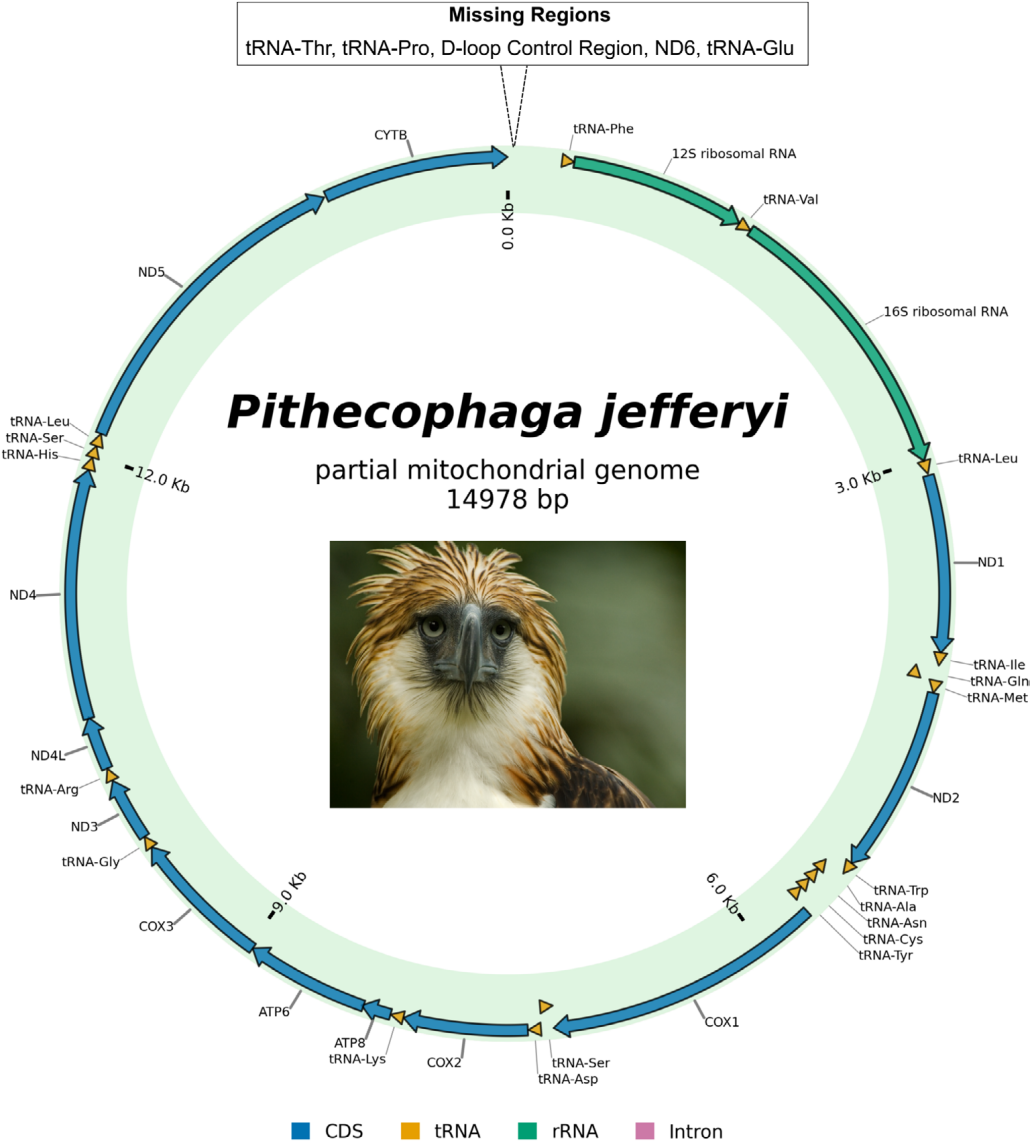
Circos plot of the partial mitochondrial genome of the Philippine eagle *Pithecophaga jefferyi* PE38 which had the longest assembled contig. Protein‐coding genes are depicted by blue arrows, tRNAs in yellow arrows, and the rRNAs in green arrows. The direction of each arrow indicates the transcriptional orientation and the strand (heavy or light) on which the gene is encoded. The missing regions are from the one contiguous gap based on the synteny with the *S. cheela* mitogenome. A representative image of the species is shown (photographed by Klaus Nigge).

A single contig which covers the partial mitochondrial genome sequence was obtained for each of the 32 samples sequenced in this study. The assemblies ranged from 13,221 bp to 14,978 bp in length, and a coverage or read depth of 9494× to 39,820×. All partial mitogenome assemblies had a GC content of 48%. Twelve (12) out of the thirteen (13) protein coding genes (PCGs) in the mitochondrial genome were sequenced and annotated. Out of the 12 PCGs annotated, 11 were complete while the cytochrome B (*CYTB*) gene was only sequenced partially with at least 1000 bp length for all 32 samples. The NADH dehydrogenase subunit 6 (*ND6*) gene was missing in all samples. Eleven (11) samples had 17 out of 22 tRNAs annotated (except Thr, Pro, Glu, Phe and Val), 20 samples had 18 of 22 tRNAs annotated (except Thr, Pro, Glu and Phe), and 1 sample had 19 tRNAs annotated (except Thr, Pro and Glu). One (1) sample, PE38, had a complete 12S and 16S rRNA sequence, and two samples, PE10 and PE33, had a partial 12S rRNA sequence (at least 500 bp) and a complete 16S rRNA sequence. The remaining 29 samples all had missing 12S rRNA sequences, 18 of which had a complete 16S rRNA sequence, while the remaining 11 samples only had a partial 16S rRNA sequence of at least 1200 bp (Table A4). In all 32 samples, the D‐loop control region was missing in the assembled contig. Nonetheless, all the PCGs, tRNAs and rRNAs that were assembled and annotated, showed synteny (Figure 2) relative to the mitogenome of the closely related species *S. cheela* (NC_015887.1), with the missing mitogenome elements all coming from a single contiguous gap which was supposedly amplified by the primers PEmtDNA 1FD and 1RS (Table A2). BLASTn analysis of the sample PE38 contig, which was the longest assembly at 14978 bp, showed that the top hits from the NCBI database are the *S. cheela* complete mitogenome (NC_015887.1) with a query cover of 98% and a percent identity of 89.09%, the *Circaetus pectoralis* mitogenome (NC_052805.1) with a query cover of 98% and a percent identity of 88.95%, and the *Aegypius monachus* mitogenome (KF682364.1) with a query cover of 98% and a percent identity of 88.63% (Table A5).

### 3.2 High Haplotype Diversity but Very Low Nucleotide Diversity in Philippine Eagles

A 13219 bp complete alignment with no gaps was generated for the 32 samples sequenced, covering 12 protein‐coding genes, 17 tRNAs and partial 16S rRNA sequence (at least 1200 bp). Using this sequence alignment, a total of 33 polymorphic sites and 35 unique mutations (base substitution) were identified. Eighteen (18) out of the 33 polymorphic sites were singletons, while the remaining 15 were parsimony informative sites. Our analysis showed a very low overall nucleotide diversity (*π*) of 0.00054 ± 0.0003 and a high overall haplotype diversity (h) of 0.9335 ± 0.024 (Table 1). A total of 17 unique haplotypes were identified from the 32 samples sequenced in this study. Comparison of the wild captives and the PEC captive‐bred eagles showed similar low nucleotide diversity (0.00056 ± 0.00005, 0.00054 ± 0.0003) and high haplotype diversity (0.947 ± 0.033, 0.9335 ± 0.024) respectively, with each group having the same total of 35 unique mutations and 33 polymorphic sites (Table 1). The wild captive subset of 19 eagles had 21 singletons and 12 parsimony informative sites. In contrast, the subset of PEC captive‐bred eagles had 18 singletons and 15 parsimony informative sites. Finally, only 13 out of the 17 total haplotypes were present in the subset of wild captives, while the subset of PEC captive‐bred eagles carries all 17 haplotypes (Table 1).

**TABLE 1:** Mitochondrial DNA polymorphism in Philippine eagles captured in the wild and those bred in captivity at the Philippine Eagle Center.

	Overall (*n* = 32)	Wild captives (*n* = 19)	PEC captive‐bred (*n* = 13)
Nucleotide diversity	0.00054 ± 0.0003	0.00056 ± 0.00005	0.00054 ± 0.0003
Haplotype diversity	0.9335 ± 0.02400	0.947 ± 0.033	0.9335 ± 0.0240
No. of haplotypes	17	13	17
No. of unique mutations	35	35	35
Total polymorphic sites	33	33	33
No. of polymorphic sites (Singletons)	18	21	18
No. of polymorphic sites (Parsimony informative)	15	12	15

### 3.3 Haplotype Network Analysis Elucidated the Maternal Lineage of 32 Philippine Eagles

**FIGURE 3:**
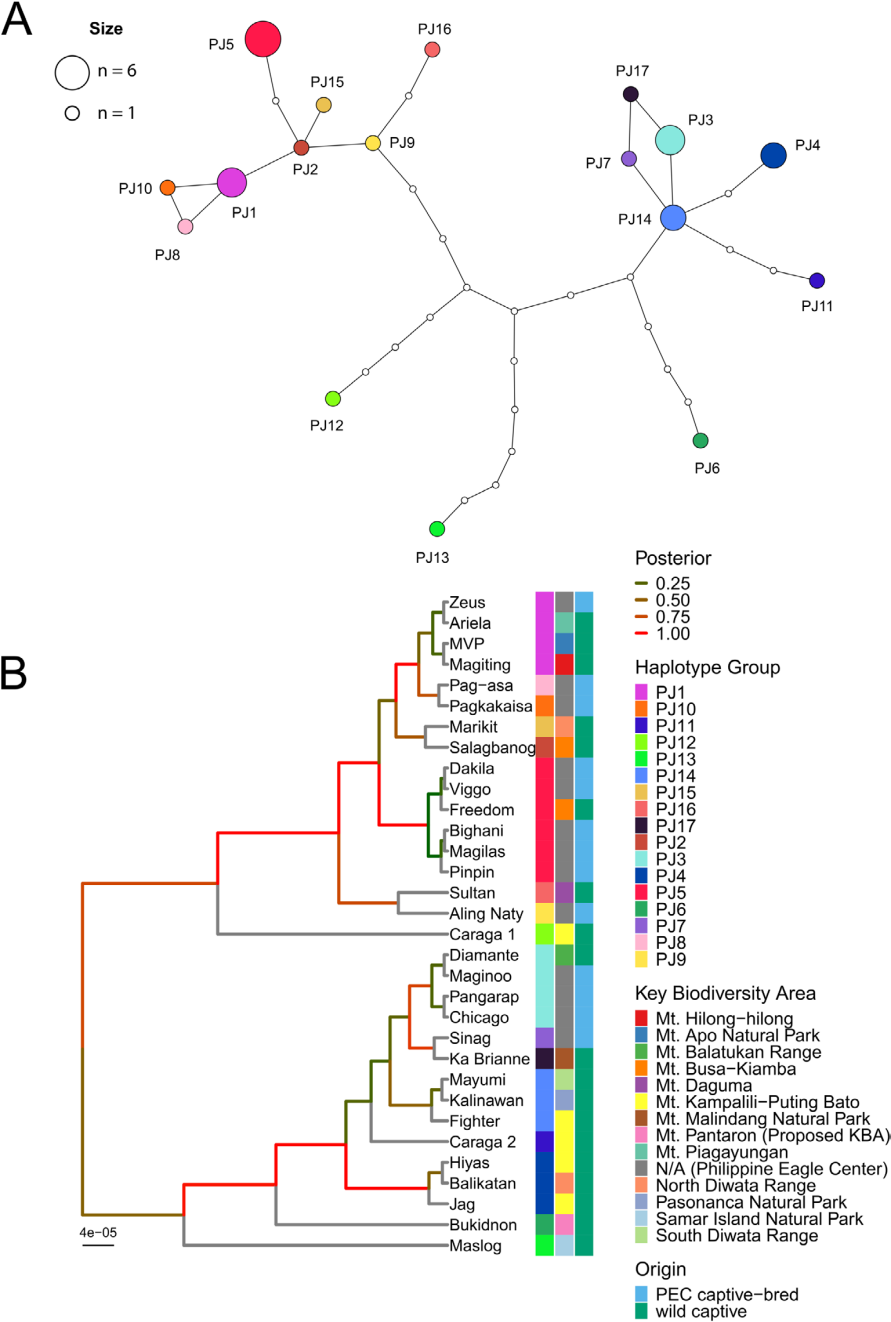
(A) Network analysis of the 17 unique haplotypes from the 32 Philippine eagles sequenced in this study. The network was constructed using the TCS method with the colored nodes representing the Philippine eagle individuals and the colorless nodes representing the mutational path or unsampled haplotypes, with each node distance equivalent to one mutation. The sizes of the colored nodes were scaled based on the number of Philippine eagles that are known to belong to that haplotype group. (B) Unrooted tree of the 32 Philippine eagles generated in BEAST with the HKY + G DNA substitution model using the 13,219 bp partial mitogenome alignment with 33 polymorphic sites. The tree branches are colored by the posterior values and a heatmap shows the eagles' origin and key biodiversity area as well as their haplotype group. The scale bar indicates the average substitution per site.

**FIGURE A2:**
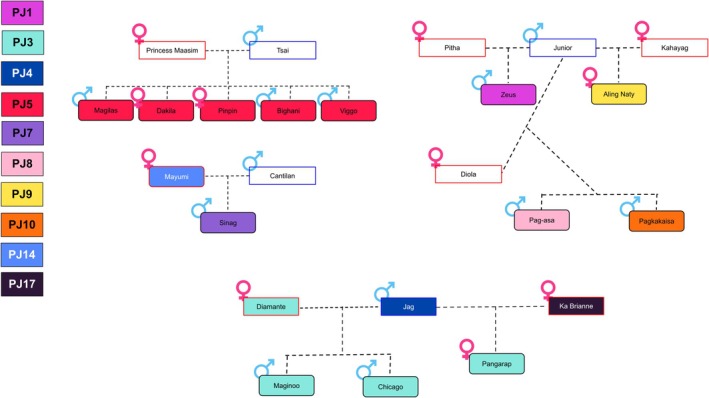
Family tree of the Philippine eagles bred in captivity at the Philippine Eagle Center (PEC) in Davao City, Philippines. The haplotype groups are indicated in colored boxes. Uncolored boxes represent unknown haplotypes of parents from the wild and PEC.

**FIGURE A3:**
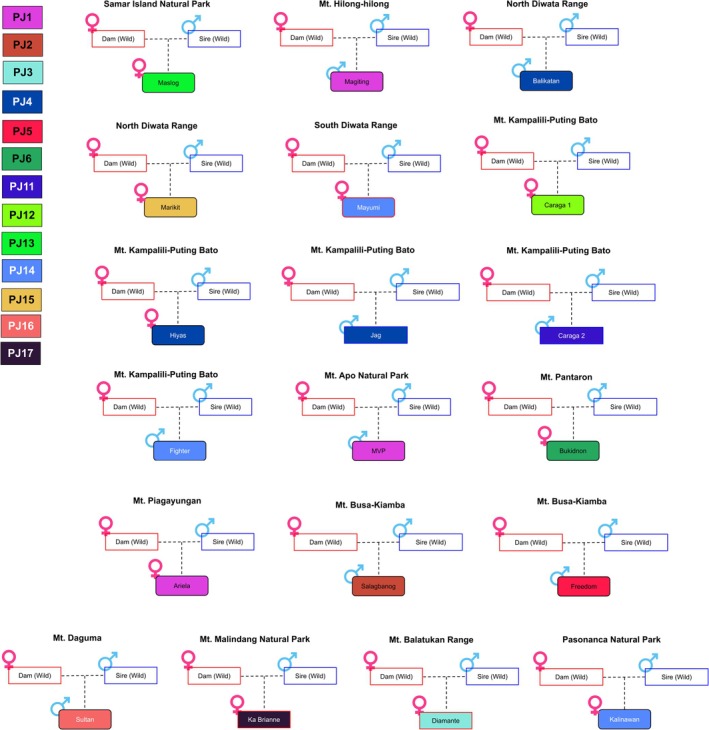
Philippine eagles rescued from the wild and rehabilitated at the Philippine Eagle Center (PEC) in Davao City, Philippines. The haplotype groups are indicated in colored boxes. Uncolored boxes represent unknown haplotypes of parents from the wild and PEC.

A haplotype network was constructed using the 33 polymorphic sites from the 13,219 bp partial mitochondrial genome alignment of 32 Philippine eagles. A total of 17 unique haplotypes were detected: 12 of which (PJ2, PJ6–PJ13, PJ15–PJ17) were each found in a single individual; two haplotypes (PJ4, PJ14), with three individuals each, two haplotypes (PJ1, PJ3) with four individuals each, and one haplotype (PJ5) represented by six individuals (Figure 3A, Figures A2, A3, Table A6). Haplotype PJ5 includes Bighani, Dakila, Magilas, Pinpin and Viggo, all PEC captive‐bred eagles from the same parents (Dam = Princess Maasim, Sire = Tsai), and a wild captive eagle Freedom from Mt. Busa–Kiamba (Figures A2, A3, Table A6). Haplotype PJ1 includes the PEC captive‐bred eagle Zeus, born from the eagles Pitha (Dam) and Junior (Sire), and three wild captive eagles Magiting, Ariela and MVP, which originated from Mt. Hilong‐hilong, Mt. Piagayungan and Mt. Apo Natural Park, respectively. Haplotype PJ3 includes the wild captive eagle Diamante from Mt. Balatukan Range, together with its two offspring with Jag (Sire), namely the eagles Maginoo and Chicago (Figures A2, A3, Table A6). Notably, PJ3 also includes another PEC captive‐bred eagle born from Jag (Sire) and Ka Brianne (Dam). Haplotype PJ4 includes three wild captive eagles Jag and Hiyas from Mt. Kampalili–Puting Bato, and another wild captive eagle Balikatan from North Diwata Range. Haplotype PJ14 also includes three wild captive eagles namely Kalinawan from Pasonanca Natural Park, Mayumi from South Diwata Range, and Fighter from Mt. Kampalili–Puting Bato (Figures A2, A3, Table A6).

Two notable genetic clusters based on the haplotype groups were observed; the first cluster consists of the haplotype groups PJ3, PJ4, PJ7, PJ11, PJ14, and PJ17, and the second cluster consists of the haplotype groups PJ1, PJ2, PJ5, PJ8, PJ9, PJ10, PJ15, and PJ16. In both clusters, a sampled haplotype group is present within one to three mutational steps (Figure 3A). The remaining haplotype groups PJ6 (Bukidnon, Mt. Pantaron), PJ12 (Caraga 1, Mt. Kampalili‐Puting Bato) and PJ13 (Maslog, Samar Island Natural Park) represent genetically distant haplotypes relative to the two clusters (Figure 3A). Phylogenetic analysis corroborates the presence of two major clades in the unrooted Bayesian phylogenetic tree (Figure 3B). While most KBAs were represented only by a single individual in this study, three KBAs namely Mt. Busa‐Kiamba, Mt. Kampalili‐Puting Bato, and North Diwata Range were represented by two, five, and two eagles, respectively. The eagles in these three KBAs were also observed to belong to at least two distinct lineages based on the haplotype network and phylogenetic tree (Figure 3B).

### 3.4 Low to Moderate Genetic Differentiation in Philippine Eagles Across Different Key Biodiversity Areas

**FIGURE 4:**
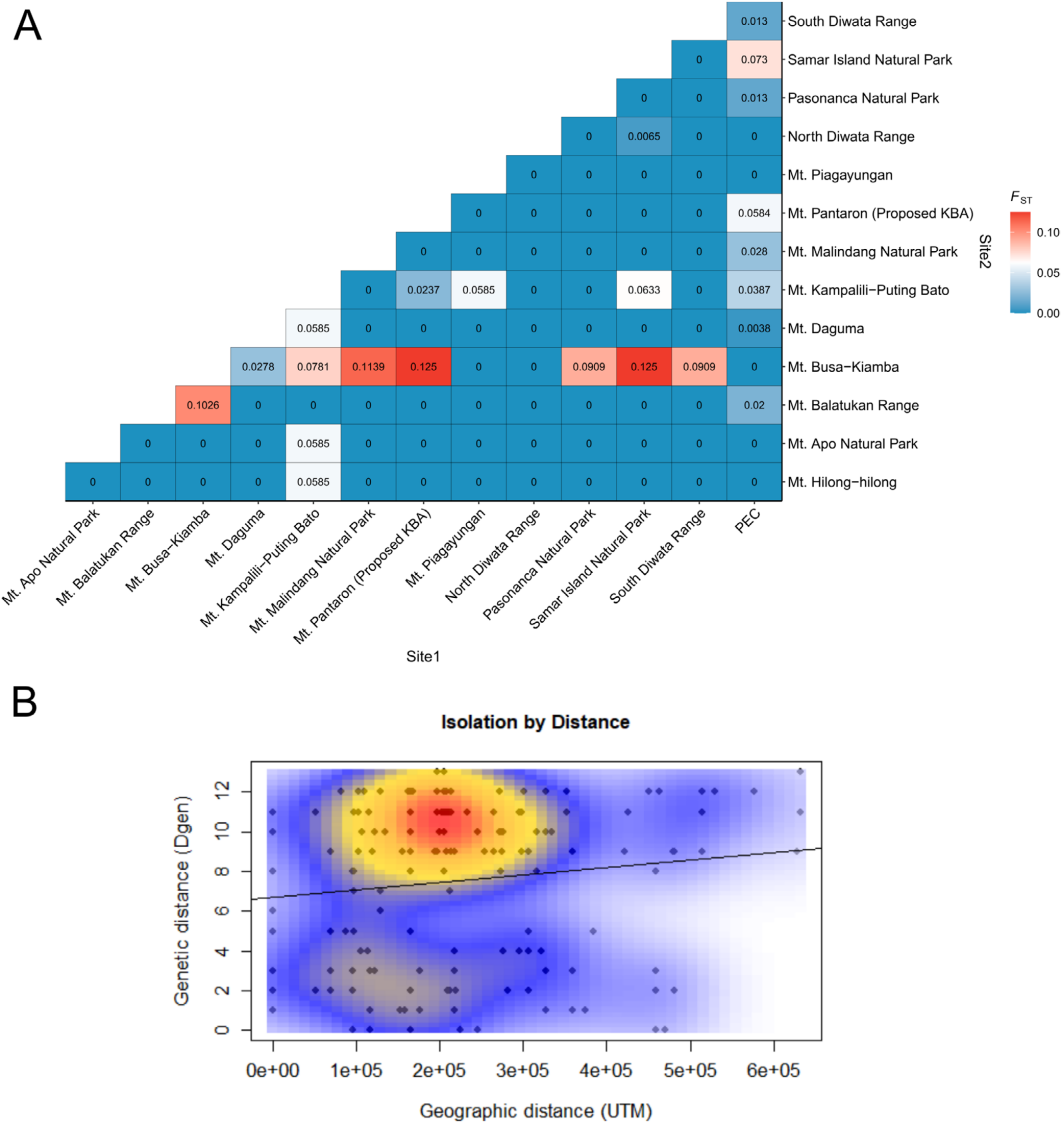
(A) *F*_ST_ values for each Philippine eagle subpopulation based on the key biodiversity area (KBA) of origin calculated using the Nei87 method in the *hierfstat* package in R. The Philippine eagles bred in captivity were grouped in one subpopulation (PEC). (B) Isolation by distance plot of the 19 Philippine eagles from the wild estimated using the *adegenet* package in R.

We defined genetic differentiation according to the *F*_*ST*_ criterion suggested by Wright (1978): *F*_*ST*_ < 0.05 indicates low genetic differentiation; 0.05 to 0.15 as moderate; 0.15–0.25 as high, and > 0.25 as very high genetic differentiation. Pairwise *F*_*ST*_ calculations among Philippine eagle subpopulations revealed relatively low values (< 0.05) across most of the KBA dyads (Figure 4A). However, we identified a few KBA dyads with moderate *F*_ST_ values (0.05 to 0.15), such as the eagles from Mt. Busa‐Kiamba‐Mt. Pantaron and Mt. Busa‐Kiamba‐Samar Island Natural Park, both with an *F*_*ST*_ value of 0.125, the highest among all the KBA dyads examined in this study. Other KBA dyads with moderate *F*_ST_ values include the following: Mt. Busa‐Kiamba‐Mt. Malindang Natural Park (0.1139), Mt. Busa‐Kiamba‐Mt. Balatukan Range (0.1026), Mt. Busa‐Kiamba‐Pasonanca Natural Park (0.0909), Mt. Busa‐Kiamba‐South Diwata Range (0.0909), Mt. Busa‐Kiamba‐Mt. Kampalili‐Puting Bato (0.0781), Samar Island Natural Park‐PEC (0.073), and Mt. Kampalili‐Puting Bato‐Samar Island Natural Park (0.0633). The Mt. Kampalili‐Puting Bato dyads with Mt. Apo Natural Park, Mt. Daguma, Mt. Piagayungan and Mt. Hilong‐hilong KBA all had moderate *F*_ST_ values at 0.0585. Additionally, the Mt. Pantaron‐PEC dyad also had a moderate *F*_ST_ value at 0.0584. Isolation by distance analysis for the 19 individuals rescued from the wild and attributed to different KBAs (excluding the PEC captive‐breds) indicated a positive but weak and nonsignificant Mantel correlation coefficient (*r*) of 0.134 (*p* = 0.116). Nevertheless, the KDE plot shows two separate density patches (Figure 4B) that represent two distinct subpopulations in the wild, consistent with the haplotype network and phylogenetic analysis.

### 3.5 Philippine Eagles Belong to the Subfamily Circaetinae

**FIGURE 5:**
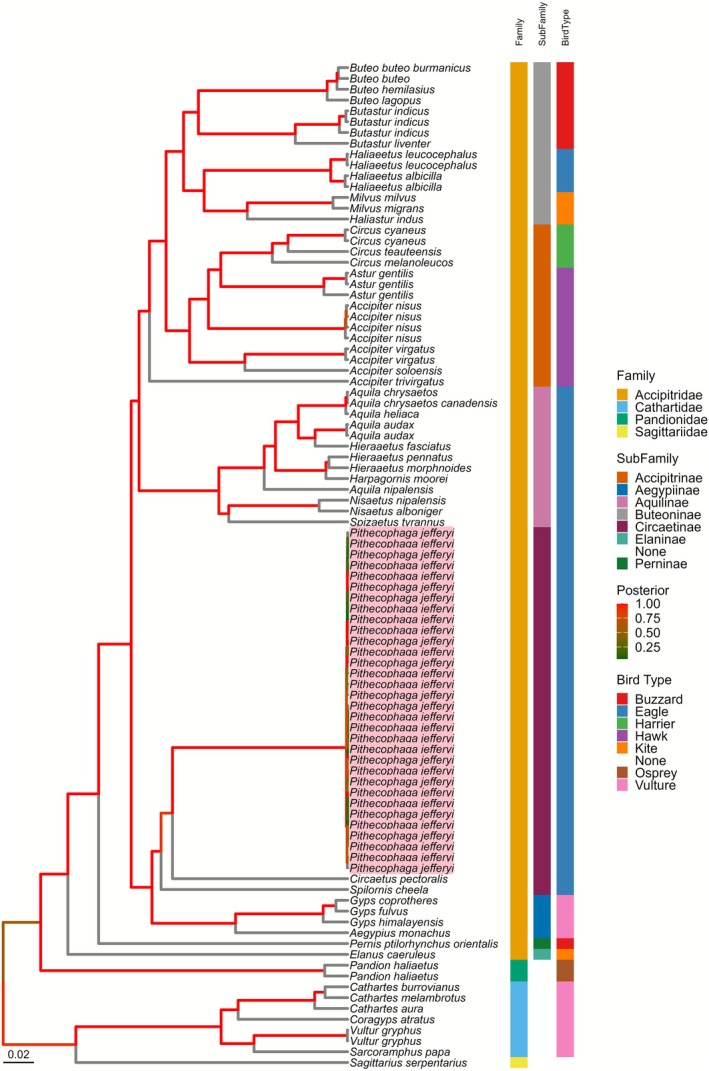
Bayesian phylogenetic analysis of birds of prey from the family Accipitridae (order Accipitriformes) and including 32 Philippine eagle samples with tip labels highlighted in pink. The final alignment used was 10,793 bp without gaps from 12 protein‐coding genes. The phylogenetic tree was generated in BEAST v.1.10.4 using the GTR + G + I DNA substitution and site heterogeneity model. Branch color indicates the posterior support value and tips are labeled with the bird species. The first heatmap after the tip labels represents the family taxa, followed by the subfamily taxa and finally the type of bird. The tree was rooted using an outgroup from the family Sagittaridae, Cathartidae, and Pandionidae which belong to the same order Accipitriformes.

A phylogenetic tree using 12 protein‐coding genes was generated to elucidate the evolutionary relationship of member species in the family Accipitridae. The black‐winged kite *Elanus caeruleus* (Desfontaines, 1789) diverged first from the rest of the Accipitridae, followed by the divergence of the species *Pernis ptilorhynchus* (Temminck, 1821) or crested honey buzzard (Figure 5). The remaining species then formed two major clades, the first one composed of species from the subfamily Aegypiinae and Circaetinae, and the second clade composed of species from the subfamily Aquilinae, Accipitrinae, and Buteoninae. *Pithecophaga jefferyi* clustered within the subfamily Circaetinae together with the species *Spilornis cheela* and *Circaetus pectoralis* . The subfamily Accipitrinae showed non‐monophyly due to the early split of *Accipiter trivirgatus* (Temminck, 1824) (MK953813) from the clade containing all other Accipitrinae and Buteoninae species, which also rendered the genus *Accipiter* non‐monophyletic (Figure 5). In contrast, the subfamily Aegypiinae, Circaetinae, Aquilinae, and Buteoninae were monophyletic. At the level of genera, *Aquila* and *Hieraaetus* were observed to be paraphyletic with the separation of *Aquila nipalensis* (MK860035) from the clade containing the other *Aquila* and *Hieraaetus* species, while *Hieraaetus fasciatus* (KP329567) clustered with the clade containing *Aquila audax* . All of these observations were supported by a strong posterior value ranging from 0.8 to 1.0. Finally, the commonly used bird types do not reflect a shared genetic relationship based on mitochondrial protein‐coding genes (Figure 5).

## 4 Discussion

Analysis of the 32 Philippine eagle partial mitogenome sequences indicated a very low overall nucleotide diversity (*π*) of 0.00054 ± 0.0003. This value is much lower compared to what was previously reported for the species using a 1132 bp sequence from the mitochondrial control region (*π* = 0.006194 ± 0.003372) (Luczon et al. 2014). Although only 32 individuals were included, this is roughly 4% of the estimated 784 individuals remaining in the wild (Sutton et al. 2023), indicating a good representation. Most threatened bird species (Vulnerable, Endangered and Critically Endangered) identified by the IUCN Red List were found to have significantly lower genetic diversity compared to non‐threatened species (Near‐Threatened, Least Concern) (Canteri et al. 2021). Specifically, the *CYTB* gene of threatened birds has on average a *π* of 0.4, and a lower range of *π* between 0.10 and 0.15 (Canteri et al. 2021). Using this as a benchmark, the overall mitochondrial nucleotide diversity for eagles in this study is more than a hundred‐fold smaller. Our results thus reinforce the status of the Philippine eagle as a critically endangered species.

Comparable nucleotide and haplotype diversity was observed between wild captives (*n* = 19) and eagles bred in captivity (*n* = 13), further highlighted by the same number of unique mutations and polymorphic sites for each population subset. However, only 13 haplotypes were identified in the wild captives, whereas 17 haplotypes were detected in the subset of eagles bred in captivity. This result suggests that more haplotype combinations are produced by the captive‐breeding efforts at PEC compared to those coming from pairs in the wild. Furthermore, this result could also mean that the captive breeding efforts employed at PEC can be used to somehow maintain or improve the genetic diversity of the species. Several studies have noted that progenies of captive breeding programs are often characterized by lower genetic diversity with potential detrimental effects on generational fitness (Posso‐Pelaez et al. 2018; Purohit et al. 2021; Thintip et al. 2021). However, other captive breeding programs maintain the genetic diversity of captive populations relative to the remaining wild population. Specifically, captive‐breeding programs contributed to population rebound in the case of the endemic pink pigeon *Nesoenas mayeri* (Prévost, 1843), the California Condor *Gymnogyps californianus* , the endangered Mauritius Kestrel *Falco punctatus* , and the Peregrine Falcon *Falco peregrinus* (Buckley et al. 2024; Campos et al. 2021; Cassin‐Sackett et al. 2021; Jackson et al. 2022; Morrison et al. 2020; Nicoll et al. 2021; Tordoff and Redig 2001; Walters et al. 2010). Hence, the ability to identify genetic groups among captive populations can help design pairing schemes to avoid inbreeding and ensure that genetic diversity is maintained for the resulting progeny (Jansamut et al. 2024; Purohit et al. 2021). Our results therefore provide important genetic data that can guide future pairing schemes for the Philippine eagle captive‐breeding program.

We identified three haplotypes that appear to be genetically distant from the rest of the samples forming two major haplotype clusters: the eagle Maslog from Eastern Samar Natural Park (PJ13), the eagle Bukidnon from Mt. Pantaron (PJ6), and the eagle Caraga 1 from Mt. Kampalili‐Puting Bato (PJ13). Eastern Samar Natural Park lies on a separate island from Mindanao, whereas Mt. Pantaron and Mt. Kampalili‐Puting Bato are situated in Central and Southern Mindanao, respectively. The genetic divergence of the eagle Maslog from other eagles is therefore consistent with geographic isolation (Hailer et al. 2015; Ogden et al. 2015). In contrast, the eagle Bukidnon and Caraga 1 may represent distinct maternal lineages that are either underrepresented in the current captive population at the PEC, or a maternal lineage potentially at risk of extinction. Nevertheless, the genetic divergence of these three individuals from other eagles suggests that these can be prioritized for breeding. Although *F*_*ST*_ analysis showed predominantly low genetic differentiation in Philippine eagles from different KBAs in the Greater Mindanao Faunal Region, the Mt. Busa‐Kiamba KBA dyads with Samar Island Natural Park, Mt. Pantaron and Mt. Kampalili‐Puting Bato showed moderate genetic differentiation. Thus, our results further highlight the availability of genetically distinct individuals or subpopulations that can be prioritized in the current captive‐breeding program to minimize inbreeding and mitigate potential deleterious consequences (Sato et al. 2017).

Philippine eagles are territorial and require, on average, more than 7000 ha of forest for each mating pair. Although suitable forest habitats have been mapped across the Philippines, only an estimated 392 potentially breeding pairs can be supported (Sutton et al. 2023). Of these, the estimated 1.7 million hectares of forest in Mindanao can support approximately 233 pairs (Sutton et al. 2023). Consistent with a predominantly low genetic differentiation, isolation by distance analysis showed a weak positive correlation, although not statistically significant, between the geographic distance and genetic distance of Philippine eagles. This result suggests a possible historical connectivity of these populations based on maternal ancestry, or that the female population undergoes occasional dispersal across large distances and between key biodiversity areas in Mindanao. Alternatively, this finding may indicate that geographic separation exerts only a minor influence on the species' genetic differentiation. Considering the limited utility of mitochondrial genetic diversity in assessing population inbreeding, future studies should incorporate nuclear markers to assess the full extent of gene flow across the eagle's population. Nevertheless, the protection of the rainforest habitats should be among the top conservation priorities, as these habitats are vital to support population dispersal and genetic diversity, as well as sustain a viable breeding population.

In addition to genetic conservation, mitochondrial genomes can also be used to assess the evolutionary relationship of avian species through phylogenetic analysis (Barker 2014; Pacheco et al. 2011). Currently, there is no consensus on the phylogenetic relationships of the members of the Neornithes or modern birds (Mayr and Clarke 2003; Pacheco et al. 2011; Springer and Gatesy 2024). A recent study showed a high level of non‐monophyly within the currently recognized genus *Accipiter* in the subfamily Accipitrinae of the order Accipitriformes (Catanach et al. 2024). Results from our phylogenetic analysis using 12 protein‐coding genes corroborate the findings of Catanach et al. (2024) which show that the Philippine eagle belongs to the monophyletic clade of the subfamily Circaetinae based on mitochondrial (e.g., legacy markers) and nuclear genes (e.g., ultraconserved elements). These results contrast with a previous study by Lerner and Mindell (2005) which suggests that the subfamily Circaetinae is non‐monophyletic based on only two mitochondrial genes (*ND2*, *CYTB*) and one nuclear intron (Lerner and Mindell 2005), highlighting the benefit of multi‐gene phylogenies in clarifying evolutionary relationships. Nevertheless, the phylogenetic relationships of many avian taxa including some species of accipitrids remain unresolved (Catanach et al. 2024). While recent studies are starting to invest in the whole genome sequencing of modern birds such as for the family Accipitridae, due to the large number of extant species comprising the Neornithes, the cost needed to sequence the genomes of all member species is projected to be massive (Callaghan et al. 2021; Catanach et al. 2024; Jetz et al. 2012). In this regard, mitogenome sequencing is a promising alternative to obtain genetic information to expedite the assessment of the phylogenetic relatedness of Neornithes using molecular data.

## 5 Conclusions

Our study demonstrated weak isolation by distance and low nucleotide diversity among Philippine eagles based on partial mitogenomes. This result reinforces the eagle's conservation status as a Critically Endangered species. However, we also observed three genetically distant haplotypes and several KBA dyads with moderate genetic differentiation. These findings highlight the potential utility of these individuals to optimize the current conservation breeding program to enhance genetic diversity and improve future reintroduction of captive‐bred eagles in the wild. These results further emphasize the need to integrate genetic information in the conservation of critically endangered species such as the Philippine eagle. Finally, the results from our phylogenetic analysis corroborate the placement of the species within the subfamily Circaetinae. Altogether, we demonstrate the value of mitogenome sequencing as an alternative to whole genome sequencing in addressing concerns on population biology, evolutionary relationships, and conservation of a threatened species.

## Author Contributions

**Michael G. Bacus:** conceptualization (equal), data curation (equal), formal analysis (equal), investigation (lead), methodology (equal), supervision (equal), visualization (lead), writing – original draft (lead), writing – review and editing (equal). **Paul Lorenzo A. Gaite:** data curation (equal), investigation (supporting), methodology (equal), supervision (equal), writing – original draft (supporting). **Joan T. Acaso:** data curation (equal), investigation (supporting), methodology (equal), writing – review and editing (supporting). **Joshua M. Cambronero:** data curation (equal), investigation (supporting), methodology (equal), writing – review and editing (supporting). **Gerry N. Ramos Jr.:** data curation (equal), investigation (supporting), methodology (equal), writing – review and editing (supporting). **April Mae M. Numeron:** data curation (equal), investigation (supporting), methodology (equal), writing – review and editing (supporting). **Dominic Tadena:** methodology (equal), resources (supporting), validation (equal), writing – review and editing (supporting). **Christian C. Labrador:** data curation (equal), investigation (supporting), methodology (equal), writing – review and editing (supporting). **Mae A. Responte:** formal analysis (equal), methodology (equal), writing – review and editing (lead). **Lief Erikson D. Gamalo:** conceptualization (equal), resources (equal), writing – original draft (supporting), writing – review and editing (supporting). **Jayson C. Ibañez:** conceptualization (equal), resources (supporting), validation (equal), writing – review and editing (lead). **Lyre Anni E. Murao:** conceptualization (equal), funding acquisition (lead), resources (lead), supervision (equal), writing – review and editing (lead).

## Funding

This work was supported by the Department of Science and Technology, Philippines.

## Conflicts of Interest

The authors declare no conflicts of interest.

## Data Availability

All Philippine eagle partial mitogenome assemblies are deposited in GenBank. The relevant metadata of the Philippine eagles included in this study and the respective accession numbers are listed in Table A1. The details of the primer design used for mitogenome enrichment are listed in Table A2. All mitogenome sequences used for phylogenetic analysis are also accessible from GenBank with the accession numbers listed in Table A3. The scripts used for the analyses are available from the corresponding author upon request.

## Appendix A

**TABLE A1:** List of Philippine eagle samples included in this study and GenBank accession numbers of the partial mitogenome assemblies.

Sample ID	Sample name	Sex	Hatch date	Admission age	Origin	Key biodiversity area	Province/City	Region	Admission date	GenBank accession number
PE1	Ka Brianne	F	Unknown	ca 1 year old	Wild captive	Mt. Malindang Natural Park	Misamis Occidental	10	July 01, 1987	PX233836
PE2	Freedom	M	Unknown	ca 2–3 month old	Wild captive	Mt. Busa‐Kiamba	Sarangani	12	May 07, 2003	PX229878
PE3	Sultan	M	Unknown	Adult	Wild captive	Mt. Daguma	South Cotabato	12	August 25, 2005	PX229879
PE5	Kalinawan	F	Unknown	Unknown	Wild captive	Pasonanca Natural Park	Zamboanga del Sur	9	June 06, 2009	PX210925
PE6	Mayumi	F	Unknown	ca 1 year old	Wild captive	South Diwata Range	Surigao del Sur	13	April 13, 2007	PX229880
PE8	Jag	M	Unknown	Adult	Wild captive	Mt. Kampalili‐ Puting Bato	Davao de Oro	11	May 05, 1984	PX229883
PE9	Marikit	F	Unknown	ca 1 year old	Wild captive	North Diwata Range	Surigao del Sur	13	November 10, 1995	PX229881
PE10	Fighter	M	Unknown	ca 1 year old	Wild captive	Mt. Kampalili‐ Puting Bato	Davao Oriental	11	January 13, 2011	PX229885
PE11	Magiting	M	Unknown	ca 4 years old	Wild captive	Mt. Hilong‐hilong	Agusan del Norte	13	September 15, 2002	PX229888
PE12	Maslog	F	Unknown	ca 2–3 years old	Wild captive	Samar Island Natural Park	Eastern Samar	8	June 28, 2019	PX233837
PE13	Caraga 1	F	Unknown	ca 1–2 years old	Wild captive	Mt. Kampalili‐ Puting Bato	Davao Oriental	11	August 01, 2020	PX229882
PE14	Caraga 2	M	Unknown	ca 3–4 years old	Wild captive	Mt. Kampalili‐ Puting Bato	Davao Oriental	11	September 26, 2020	PX229884
PE15	Hiyas	F	Unknown	ca 1 year old	Wild captive	Mt. Kampalili‐ Puting Bato	Davao Oriental	11	April 07, 1999	PX229886
PE16	Bighani	M	December 07, 2007	N/A	PEC captive‐bred	N/A	Davao City	11	N/A	PX229887
PE17	Pagkakaisa	M	October 25, 1992	N/A	PEC captive‐bred	N/A	Davao City	11	N/A	PX229889
PE18	Aling Naty	F	November 11, 2000	N/A	PEC captive‐bred	N/A	Davao City	11	N/A	PX233838
PE19	Pag‐asa	M	January 15, 1992	N/A	PEC captive‐bred	N/A	Davao City	11	N/A	PX233839
PE20	Zeus	M	February 04, 2002	N/A	PEC captive‐bred	N/A	Davao City	11	N/A	PX223339
PE24	Pangarap	F	February 23, 1999	N/A	PEC captive‐bred	N/A	Davao City	11	N/A	PX223338
PE25	Sinag	M	December 07, 2015	N/A	PEC captive‐bred	N/A	Davao City	11	N/A	PX241551
PE26	Dakila	F	November 25, 2005	N/A	PEC captive‐bred	N/A	Davao City	11	N/A	PX223337
PE28	Magilas	M	January 16, 2003	N/A	PEC captive‐bred	N/A	Davao City	11	N/A	PX114337
PE29	Pinpin	F	November 23, 2006	N/A	PEC captive‐bred	N/A	Davao City	11	N/A	PX223336
PE30	Ariela	F	Unknown	ca 3–4 years old	Wild captive	Mt. Piagayungan	Lanao del Sur	BARMM	April 25, 2015	PX114338
PE31	Bukidnon	F	Unknown	ca 2–3 months	Wild captive	Mt. Pantaron	Bukidnon	10	February 26, 2021	PX114339
PE32	Viggo	M	March 07, 2010	N/A	PEC captive‐bred	N/A	Davao City	11	N/A	PX114340
PE33	Diamante	F	Unknown	ca 5 years old	Wild captive	Mt. Balatukan Range	Misamis Oriental	10	May 05, 1984	PX114341
PE34	Balikatan	M	Unknown	ca 3–4 years old	Wild captive	North Diwata Range	Surigao del Norte	13	August 28, 2020	PX210923
PE35	Maginoo	M	December 15, 2000	N/A	PEC captive‐bred	N/A	Davao City	11	N/A	PX210924
PE38	Chicago	M	December 18, 2004	N/A	PEC captive‐bred	N/A	Davao City	11	N/A	PX114333
PE39	Salagbanog	M	Unknown	ca 3–4 years old	Wild captive	Mt. Busa‐Kiamba	Sarangani	12	January 09, 2021	PX114334
PE41	MVP	M	Unknown	ca 1 year old	Wild captive	Mt. Apo Natural Park	Davao del Sur	11	April 18, 2011	PX114335

**TABLE A2:** List of long‐range PCR primers designed to amplify the Philippine eagle mitogenome and their *in silico* properties.

Primer ID	Nucleotide sequence (5′‐3′)	Position ( *S. cheela* )	Length (bp)	GC %	*T*_m_ (°C)	Hairpin (ΔG)	Self dimer (ΔG)	Cross dimer (ΔG)
PEmtDNA‐1FD	CAT GCA CTA CAC CGC AGA CAC	159	21	57.14	60.16	−2.0	−3.4	−2.4
PEmtDNA‐1RS	CTT GAA CCT CTG GGT AAA GGG	7338	21	52.38	56.18	−1.5	−1.5	
PEmtDNA‐2FD	CCA ACC GAG CTG GGT GAT AG	6030	20	60.0	57.15	−1.5	−3.0	−5.0
PEmtDNA‐2RS	GGA AGA ATG CCC AGA AGA AGC C	13,553	22	54.55	58.97	0.0	0.0	
PEmtDNA‐3FD	CAA GCC AGC CGC ATC TAA CC	11,537	20	60.0	59.75	0.0	0.0	−5.4
PEmtDNA‐3RS	GTT GGC TGC CGA TTC ATG TGA G	1027	22	60.0	59.75	0.0	−2.4	

**TABLE A3:** List of mitogenome sequences from the order Accipitriformes obtained from GenBank.

Accession ID	Species	Genus	Family	Subfamily	BirdType	CommonName	Mitogenome size (bp)
OK662584	*Elanus caeruleus*	Elanus	Accipitridae	Elaninae	Kite	Black‐winged kite	18,898
LC541458	*Pernis ptilorhynchus orientalis*	Pernis	Accipitridae	Perninae	Buzzard	Crested honey buzzard	18,340
JN191388	*Spilornis cheela*	Spilornis	Accipitridae	Circaetinae	Eagle	Crested serpent eagle	18,291
MN356306	*Circaetus pectoralis*	Circaetus	Accipitridae	Circaetinae	Eagle	Black‐chested snake eagle	17,473
KX893247	*Gyps fulvus*	Gyps	Accipitridae	Aegypiinae	Vulture	Eurasian griffon vulture	18,094
KY594709	*Gyps himalayensis*	Gyps	Accipitridae	Aegypiinae	Vulture	Himalayan vulture	17,381
MF683387	*Gyps coprotheres*	Gyps	Accipitridae	Aegypiinae	Vulture	Cape vulture	16,908
KF682364	*Aegypius monachus*	Aegypius	Accipitridae	Aegypiinae	Vulture	Cinereous vulture	17,811
MT319112	*Aquila chrysaetos canadensis*	Aquila	Accipitridae	Aquilinae	Eagle	Golden eagle	17,472
KF905228	*Aquila chrysaetos*	Aquila	Accipitridae	Aquilinae	Eagle	Golden eagle	17,332
MK860035	*Aquila nipalensis*	Aquila	Accipitridae	Aquilinae	Eagle	Steppe eagle	18,450
KU646835	*Aquila heliaca*	Aquila	Accipitridae	Aquilinae	Eagle	Eastern imperial eagle	18,067
MZ595097	*Aquila audax*	Aquila	Accipitridae	Aquilinae	Eagle	Wedge‐tailed eagle	17,324
MK453378	*Aquila audax*	Aquila	Accipitridae	Aquilinae	Eagle	Wedge‐tailed eagle	17,284
AP008239	*Nisaetus alboniger*	Nisaetus	Accipitridae	Aquilinae	Eagle	Blyth's hawk‐eagle	17,977
AP008238	*Nisaetus nipalensis*	Nisaetus	Accipitridae	Aquilinae	Eagle	Mountain hawk‐eagle	17,667
MN356300	*Spizaetus tyrannus*	Spizaetus	Accipitridae	Aquilinae	Eagle	Black hawk‐eagle	17,479
KP329567	*Hieraaetus fasciatus*	Hieraaetus	Accipitridae	Aquilinae	Eagle	Bonelli's eagle	18,513
MK294165	*Hieraaetus pennatus*	Hieraaetus	Accipitridae	Aquilinae	Eagle	Booted eagle	17,666
MK294164	*Hieraaetus morphnoides*	Hieraaetus	Accipitridae	Aquilinae	Eagle	Little eagle	17,659
MK294166	*Harpagornis moorei*	Hieraaetus	Accipitridae	Aquilinae	Eagle	New Zealand giant eagle	17,272
KJ680300	*Accipiter nisus*	Accipiter	Accipitridae	Accipitrinae	Hawk	Eurasian sparrowhawk	19,417
KM360148	*Accipiter nisus*	Accipiter	Accipitridae	Accipitrinae	Hawk	Eurasian sparrowhawk	18,647
MN929010	*Accipiter nisus*	Accipiter	Accipitridae	Accipitrinae	Hawk	Eurasian sparrowhawk	18,352
MN122826	*Accipiter nisus*	Accipiter	Accipitridae	Accipitrinae	Hawk	Eurasian sparrowhawk	16,426
AP010797	*Accipiter gentilis*	Accipiter	Accipitridae	Accipitrinae	Hawk	Northern goshawk	18,266
MN122867	*Accipiter gentilis*	Accipiter	Accipitridae	Accipitrinae	Hawk	Northern goshawk	16,197
PQ049665	*Accipiter gentilis*	Accipiter	Accipitridae	Accipitrinae	Hawk	Northern goshawk	16,197
MK953813	*Accipiter trivirgatus*	Accipiter	Accipitridae	Accipitrinae	Hawk	Crested goshawk	18,454
KJ699124	*Accipiter virgatus*	Accipiter	Accipitridae	Accipitrinae	Hawk	Besra sparrowhawk	17,952
KP336714	*Accipiter virgatus*	Accipiter	Accipitridae	Accipitrinae	Hawk	Besra sparrowhawk	17,654
KJ680303	*Accipiter soloensis*	Accipiter	Accipitridae	Accipitrinae	Hawk	Chinese sparrowhawk	17,900
KU237286	*Circus cyaneus*	Circus	Accipitridae	Accipitrinae	Harrier	Hen harrier	20,173
KX925606	*Circus cyaneus*	Circus	Accipitridae	Accipitrinae	Harrier	Hen harrier	18,754
MK386464	*Circus teauteensis*	Circus	Accipitridae	Accipitrinae	Harrier	Eyles's harrier	17,873
KT438620	*Circus melanoleucos*	Circus	Accipitridae	Accipitrinae	Harrier	Pied harrier	17,749
MG930481	*Milvus migrans*	Milvus	Accipitridae	Buteoninae	Kite	Black kite	18,016
MN122837	*Milvus milvus*	Milvus	Accipitridae	Buteoninae	Kite	Red kite	17,883
OP133375	*Haliastur indus*	Haliastur	Accipitridae	Buteoninae	Kite	Brahminy kite	19,055
MN356134	*Haliaeetus leucocephalus*	Haliaeetus	Accipitridae	Buteoninae	Eagle	Bald eagle	18,645
PQ049664	*Haliaeetus leucocephalus*	Haliaeetus	Accipitridae	Buteoninae	Eagle	Bald eagle	18,645
MK043028	*Haliaeetus albicilla*	Haliaeetus	Accipitridae	Buteoninae	Eagle	White‐tailed eagle	17,719
MN356434	*Haliaeetus albicilla*	Haliaeetus	Accipitridae	Buteoninae	Eagle	White‐tailed eagle	16,462
AB830617	*Butastur liventer*	Butastur	Accipitridae	Buteoninae	Buzzard	Rufous‐winged buzzard	19,673
AB830616	*Butastur indicus*	Butastur	Accipitridae	Buteoninae	Buzzard	Gray‐faced buzzard	19,059
MW017133	*Butastur indicus*	Butastur	Accipitridae	Buteoninae	Buzzard	Gray‐faced buzzard	18,169
LC541471	*Butastur indicus*	Butastur	Accipitridae	Buteoninae	Buzzard	Gray‐faced buzzard	18,013
AF380305	*Buteo buteo*	Buteo	Accipitridae	Buteoninae	Buzzard	Common buzzard	18,674
KM364882	*Buteo buteo burmanicus*	Buteo	Accipitridae	Buteoninae	Buzzard	Himalayan buzzard	18,231
KP337337	*Buteo lagopus*	Buteo	Accipitridae	Buteoninae	Buzzard	Rough‐legged buzzard	18,559
KT935541	*Buteo hemilasius*	Buteo	Accipitridae	Buteoninae	Buzzard	Upland buzzard	18,079
MN356325	*Pandion haliaetus*	Pandion	Pandionidae	None	Osprey	Osprey	18,153
DQ780884	*Pandion haliaetus*	Pandion	Pandionidae	None	Osprey	Osprey	17,864
KF961184	*Sagittarius serpentarius*	Sagittarius	Sagittariidae	None	None	Secretary bird	16,773
MN720441	*Cathartes burrovianus*	Cathartes	Cathartidae	None	Vulture	Lesser yellow‐headed vulture	19,285
PQ153910	*Cathartes melambrotus*	Cathartes	Cathartidae	None	Vulture	Greater yellow‐headed vulture	19,232
AY463690	*Cathartes aura*	Cathartes	Cathartidae	None	Vulture	Turkey vulture	16,779
MN720440	*Coragyps atratus*	Coragyps	Cathartidae	None	Vulture	Black vulture	19,329
MN720442	*Sarcoramphus papa*	Sarcoramphus	Cathartidae	None	Vulture	King vulture	19,302
MN720444	*Vultur gryphus*	Vultur	Cathartidae	None	Vulture	Andean condor	19,287
MZ223429	*Vultur gryphus*	Vultur	Cathartidae	None	Vulture	Andean condor	16,808

**TABLE A4:** Assembly statistics of partial mitogenome sequences of Philippine eagles.

Sample ID	Assembly length (bp)	Coverage or read depth (X)	GC content	Protein‐coding genes (PCG)			Ribosomal RNA (rRNA)		Missing transfer RNA (tRNA)
				Number of complete sequence (> 99% length)	Partial sequence	Missing (less than 50 bp cover)	16S rRNA	12S rRNA	
PE1	13,225	11,917	48%	11	CYTB	ND6	Partial	Missing	Thr, Pro, Glu, Phe, Val
PE2	13,662	15,358	48%	11	CYTB	ND6	Complete	Missing	Thr, Pro, Glu, Phe
PE3	13,660	12,537	48%	11	CYTB	ND6	Complete	Missing	Thr, Pro, Glu, Phe
PE5	14,167	19,285	48%	11	CYTB	ND6	Complete	Missing	Thr, Pro, Glu, Phe
PE6	13,258	24,688	48%	11	CYTB	ND6	Partial	Missing	Thr, Pro, Glu, Phe, Val
PE8	14,170	13,881	48%	11	CYTB	ND6	Complete	Missing	Thr, Pro, Glu, Phe
PE9	13,234	16,365	48%	11	CYTB	ND6	Partial	Missing	Thr, Pro, Glu, Phe, Val
PE10	14,179	18,203	48%	11	CYTB	ND6	Complete	Partial	Thr, Pro, Glu, Phe
PE11	13,660	9494	48%	11	CYTB	ND6	Complete	Missing	Thr, Pro, Glu, Phe
PE12	13,658	27,784	48%	11	CYTB	ND6	Complete	Missing	Thr, Pro, Glu, Phe
PE13	13,220	29,282	48%	11	CYTB	ND6	Partial	Missing	Thr, Pro, Glu, Phe, Val
PE14	13,658	18,911	48%	11	CYTB	ND6	Complete	Missing	Thr, Pro, Glu, Phe
PE15	13,660	20,585	48%	11	CYTB	ND6	Complete	Missing	Thr, Pro, Glu, Phe
PE16	13,661	21,193	48%	11	CYTB	ND6	Complete	Missing	Thr, Pro, Glu, Phe
PE17	13,220	27,642	48%	11	CYTB	ND6	Partial	Missing	Thr, Pro, Glu, Phe, Val
PE18	14,168	25,157	48%	11	CYTB	ND6	Complete	Missing	Thr, Pro, Glu, Phe
PE19	13,225	28,476	48%	11	CYTB	ND6	Partial	Missing	Thr, Pro, Glu, Phe, Val
PE20	13,667	17,197	48%	11	CYTB	ND6	Complete	Missing	Thr, Pro, Glu, Phe
PE24	13,221	30,373	48%	11	CYTB	ND6	Partial	Missing	Thr, Pro, Glu, Phe, Val
PE25	13,224	22,326	48%	11	CYTB	ND6	Partial	Missing	Thr, Pro, Glu, Phe, Val
PE26	13,659	16,481	48%	11	CYTB	ND6	Complete	Missing	Thr, Pro, Glu, Phe
PE28	13,666	39,820	48%	11	CYTB	ND6	Complete	Missing	Thr, Pro, Glu, Phe
PE29	13,220	15,746	48%	11	CYTB	ND6	Partial	Missing	Thr, Pro, Glu, Phe, Val
PE30	13,221	24,847	48%	11	CYTB	ND6	Partial	Missing	Thr, Pro, Glu, Phe, Val
PE31	13,661	18,355	48%	11	CYTB	ND6	Complete	Missing	Thr, Pro, Glu, Phe
PE32	13,665	24,311	48%	11	CYTB	ND6	Complete	Missing	Thr, Pro, Glu, Phe
PE33	14,178	21,793	48%	11	CYTB	ND6	Complete	Partial	Thr, Pro, Glu, Phe
PE34	13,686	21,286	48%	11	CYTB	ND6	Complete	Missing	Thr, Pro, Glu, Phe
PE35	13,222	21,843	48%	11	CYTB	ND6	Partial	Missing	Thr, Pro, Glu, Phe, Val
PE38	14,978	21,729	48%	11	CYTB	ND6	Complete	Complete	Thr, Pro, Glu
PE39	13,658	27,230	48%	11	CYTB	ND6	Complete	Missing	Thr, Pro, Glu, Phe
PE41	13,660	20,316	48%	11	CYTB	ND6	Complete	Missing	Thr, Pro, Glu, Phe

**TABLE A5:** Top five NCBI‐BLAST hits of the Philippine eagle PE38 sample with the longest assembled contig for partial mitochondrial genome at 14978 bp.

Blast hit description	Query cover	*E*‐value	% Identity	Accession ID
*Spilornis cheela* mitochondrion, complete genome	98%	0	89.09	NC_015887.1
*Circaetus pectoralis* mitochondrion, complete genome	98%	0	88.95	NC_052805.1
*Aegypius monachus* mitochondrion, complete genome	98%	0	88.63	KF682364.1
*Gyps fulvus* mitochondrion, complete genome	98%	0	88.49	NC_036050.1
*Gyps coprotheres* mitochondrion, complete genome	98%	0	88.49	MF683387.1

**TABLE A6:** Philippine eagle individuals in each of the 17 haplotypes identified.

Haplotype group	Sample ID	Sample name	Origin	Key biodiversity area	Dam	Sire
PJ1	PE11	Magiting	Wild captive	Mt. Hilong‐hilong	Unknown	Unknown
	PE20	Zeus	PEC captive‐bred	N/A	Pitha	Junior
	PE30	Ariela	Wild captive	Mt. Piagayungan	Unknown	Unknown
	PE41	MVP	Wild captive	Mt. Apo	Unknown	Unknown
PJ2	PE39	Salagbanog	Wild captive	Mt. Busa‐Kiamba	Unknown	Unknown
PJ3	PE24	Pangarap	PEC captive‐bred	N/A	Ka Brianne	Jag
	PE33	Diamante	Wild captive	Mt. Balatukan Range	Unknown	Unknown
	PE35	Maginoo	PEC captive‐bred	N/A	Diamante	Jag
	PE38	Chicago	PEC captive‐bred	N/A	Diamante	Jag
PJ4	PE8	Jag	Wild captive	Mt. Kampalili‐ Puting Bato	Unknown	Unknown
	PE15	Hiyas	Wild captive	Mt. Kampalili‐ Puting Bato	Unknown	Unknown
	PE34	Balikatan	Wild captive	North Diwata Range	Unknown	Unknown
PJ5	PE2	Freedom	Wild captive	Mt. Busa‐Kiamba	Unknown	Unknown
	PE16	Bighani	PEC captive‐bred	N/A	Princess Maasim	Tsai
	PE26	Dakila	PEC captive‐bred	N/A	Princess Maasim	Tsai
	PE28	Magilas	PEC captive‐bred	N/A	Princess Maasim	Tsai
	PE29	Pinpin	PEC captive‐bred	N/A	Princess Maasim	Tsai
	PE32	Viggo	PEC captive‐bred	N/A	Princess Maasim	Tsai
PJ6	PE31	Bukidnon	Wild captive	Mt. Pantaron	Unknown	Unknown
PJ7	PE25	Sinag	PEC captive‐bred	N/A	Tinuy‐an	Cantilan
PJ8	PE19	Pag‐asa	PEC captive‐bred	N/A	Diola	Junior
PJ9	PE18	Aling Naty	PEC captive‐bred	N/A	Kahayag	Junior
PJ10	PE17	Pagkakaisa	PEC captive‐bred	N/A	Diola	Junior
PJ11	PE14	Caraga 2	Wild captive	Mt. Kampalili‐ Puting Bato	Unknown	Unknown
PJ12	PE13	Caraga 1	Wild captive	Mt. Kampalili‐ Puting Bato	Unknown	Unknown
PJ13	PE12	Maslog	Wild captive	Samar Island Natural Park	Unknown	Unknown
PJ14	PE5	Kalinawan	Wild captive	Pasonanca Natural Park	Unknown	Unknown
	PE6	Mayumi	Wild captive	South Diwata Range	Unknown	Unknown
	PE10	Fighter	Wild captive	Mt. Kampalili‐ Puting Bato	Unknown	Unknown
PJ15	PE9	Marikit	Wild captive	North Diwata Range	Unknown	Unknown
PJ16	PE3	Sultan	Wild captive	Mt. Daguma	Unknown	Unknown
PJ17	PE1	Ka Brianne	Wild captive	Mt. Malindang Natural Park	Unknown	Unknown
